# Functional and molecular characterization of suicidality factors using phenotypic and genome-wide data

**DOI:** 10.1038/s41380-022-01929-5

**Published:** 2023-01-06

**Authors:** Andrea Quintero Reis, Brendan A. Newton, Ronald Kessler, Renato Polimanti, Frank R. Wendt

**Affiliations:** 1 American University of Antigua College of Medicine, Osbourn, Antigua and Barbuda; 2 Forensic Science Program, University of Toronto, Mississauga, ON Canada; 3 Department of Health Care Policy, Harvard Medical School, Boston, MA USA; 4 Department of Psychiatry, Yale School of Medicine, New Haven, CT USA; 5 VA CT Healthcare System, West Haven, CT USA; 6 Department of Anthropology, University of Toronto, Mississauga, ON Canada; 7 Biostatistics Division, Dalla Lana School of Public Health, University of Toronto, Toronto, ON Canada

**Keywords:** Depression, Genetics

## Abstract

Genome-wide association studies (GWAS) of suicidal thoughts and behaviors support the existence of genetic contributions. Continuous measures of psychiatric disorder symptom severity can sometimes model polygenic risk better than binarized definitions. We compared two severity measures of suicidal thoughts and behaviors at the molecular and functional levels using genome-wide data. We used summary association data from GWAS of four traits analyzed in 122,935 individuals of European ancestry: *thought life was not worth living* (TLNWL), *thoughts of self-harm*, *actual self-harm*, and *attempted suicide*. A new trait for suicidal thoughts and behaviors was constructed first, phenotypically, by aggregating the previous four traits (termed “suicidality”) and second, genetically, by using genomic structural equation modeling (gSEM; termed S-factor). Suicidality and S-factor were compared using SNP-heritability (*h*^*2*^) estimates, genetic correlation (*r*_*g*_), partitioned *h*^*2*^, effect size distribution, transcriptomic correlations (*ρ*_GE_) in the brain, and cross-population polygenic scoring (PGS). The S-factor had good model fit (*χ*^2^ = 0.21, AIC = 16.21, CFI = 1.00, SRMR = 0.024). Suicidality (*h*^*2*^ = 7.6%) had higher *h*^*2*^ than the S-factor (*h*^*2*^ = 2.54, P_diff_ = 4.78 × 10^−13^). Although the S-factor had a larger number of non-null susceptibility loci (π_c_ = 0.010), these loci had small effect sizes compared to those influencing suicidality (π_c_ = 0.005, P_diff_ = 0.045). The *h*^*2*^ of both traits was enriched for conserved biological pathways. The *r*_*g*_ and *ρ*_GE_ support highly overlapping genetic and transcriptomic features between suicidality and the S-factor. PGS using European-ancestry SNP effect sizes strongly associated with TLNWL in Admixed Americans: Nagelkerke’s *R*^*2*^ = 8.56%, *P* = 0.009 (PGS_suicidality_) and Nagelkerke’s *R*^*2*^ = 7.48%, *P* = 0.045 (PGS_S-factor_). An aggregate suicidality phenotype was statistically more heritable than the S-factor across all analyses and may be more informative for future genetic study designs interested in common genetic factors among different suicide related phenotypes.

## Introduction

Death by suicide is responsible for more than 700,000 deaths per year [1]. The World Health Organization invested in global advocacy and awareness programs toward reducing stigma and increasing access to care. However, death by suicide ranks as the fourth leading cause of death among teens and young adults [2]. Twin, family, and adoption studies show a heritability (i.e., phenotypic variation explained by genetic differences) of suicidal thoughts and behaviors between 30 and 50% [3].

Large genome-wide association studies (GWAS) of individual thoughts and behaviors associated with death by suicide demonstrate small but robust heritability estimates attributed to common genetic variation (*h*^*2*^): 1.25% for attempted suicide (AS) in the Million Veteran Program (MVP) [4], 1.9–4.6% in a Danish study of AS with and without considering mental health diagnoses [5], 5.7–6.8% in two large meta-analyses of AS lead by the International Suicide Genetics Consortium [1, 6]. When considering comorbid psychiatric diagnoses, the Psychiatric Genomics Consortium has previously reported a genetic signal for AS that is independent of major depressive disorder (MDD), schizophrenia, and bipolar disorder [1]. Furthermore, the literature varies on the degree of genetic and clinical overlap between suicidal ideation, suicidal planning, non-suicidal self-injury, suicidal self-injury, suicide attempt, and death by suicide. These phenotypes are heterogeneous, and many studies support studying them as discrete items to dissect the unique genetic factors associated with each [7–10]. However, all of these trait associate with death by suicide, which warrants assessment of the genetic features shared among suicidal thoughts and behaviors. It remains unclear how these traits genetically overlap with one another and to mental health diagnoses.

Other suicidal behaviors occur well before AS and may include ideation and planning the attempt to end one’s life. Though death by suicide is not a diagnosis, these thoughts and behaviors may be modeled as a severity continuum termed ‘suicidality’ analogous to symptom severity measures used for formal DSM diagnoses. Among GWAS of psychiatric disorders and conditions associated with AS and death by suicide, continuous measures of symptom severity appear to better model polygenic risk than binarized case-control items, leading to increased statistical power in gene discovery [11, 12]. This is especially true for traits strongly correlated with suicidal thoughts and behaviors such as posttraumatic stress disorder [11] and MDD [13]. It therefore stands to reason that a suicidality measure capturing continuous variation across suicidal thoughts and behaviors would be more statistically powerful than any individual dichotomized definition. Strawbridge, et al. reported a genome-wide association study of one definition of suicidality in the UK Biobank (UKB) and reported an *h*^*2*^ of 7.6% in a sample substantially smaller than the most contemporary meta-analyses of AS [1, 4, 6, 14, 15].

Though showing higher *h*^*2*^ than individual thoughts and behaviors associated with death by suicide, aggregating these items at the phenotype level may induce phenotypic heterogeneity that limits the potential discovery of genome-wide significant loci and biological processes relevant for discrete, yet related, behaviors. A primary limitation of some recently introduced phenotypically aggregated suicidality measures is the equal contribution of each questionnaire item to the final suicidality rating regardless of the heritability of each item or the relationship between items. Genomic structural equation modeling (gSEM [16, 17]) is a multivariate method that permits building factor structure(s) that account for the heritability of each indicator and the relationship between indicators. For example, gSEM has been used previously to describe how the 10-item Alcohol Use Disorder Identification Test reflects a correlated two-factor structure of problematic use and consumption [18].

This study asked whether there is any benefit to studying a gSEM derived “S-factor” in addition to a questionnaire-derived suicidality measure to inform suicide biology. We report *h*^*2*^ differences between suicidality and S-factor and compare these trait definitions on the basis of genetic correlation with other psychopathologies and mental health diagnoses, functional enrichment underlying their *h*^*2*^ estimates, and transcriptomic signatures across various relevant brain regions. Our findings reinforce the greater statistical power of a phenotypically-derived suicidality factor and demonstrate a systematic reduction of signal in all analyses when analyzing the S-factor.

## Methods

### S-factor modeling

The gSEM method models the multivariate genetic architecture of sets of traits by incorporating genetic covariance structure into multivariate GWAS. Using gSEM [16, 17], common factor GWAS was performed on the S-factor linking four traits describing the thoughts and behaviors associated with death by suicide and range in severity from ideation to attempt to end one’s life. The four common factor indicators were questions from the UKB self-harm behaviors section of the online Mental Health Questionnaire and have been previously described by Strawbridge, et al. [15]: *thought life was not worth living* (TLNWL), *thoughts of self-harm or suicide* (TSH), *actual self-harm* (ASH), and *AS*. Note that participants could respond to ASH with “yes” for deliberate acts of self-harm whether or not they intended to end their own lives.

We hypothesized that a single factor explained the four indicator variables and as such no exploratory factor analysis was performed. Multivariable linkage disequilibrium score regression (LDSC) was used to obtain a genetic covariance and corresponding sampling matrix based on a European ancestry linkage disequilibrium reference panel reflecting the 1000 Genomes Project EUR superpopulation. All factor modeling used diagonally weighted least squares estimation and promax rotation. Four model fit statistics were evaluated: chi-squared (χ^2^), comparative fit index (CFI), Akaike information criterion (AIC), and standardized root mean square residual (SRMR). Briefly, *χ*^2^ indexes whether the modelled genetic covariance matrix differs from the empirical matrix. CFI tests whether the proposed model fits better than a model that assumes all indicators are heritable but uncorrelated. AIC measures relative model fit and can be used to compare multiple models. SRMR is a measure of approximate model fit calculated as the standardized root mean square difference between model implied and observed correlations among covariance matrices. Each fit statistic has its strengths and weaknesses, so we considered several features in deciding the best model. We declared a model superior if it had a lower AIC value, lower SRMR, and higher CFI.

### Trait description

The UKB is a population-based cohort of >500,000 participants with deep phenotyping of lifestyle factors, mental and physical health outcomes, anthropometric measurements, and other traits. Our analysis used the self-harm behavior GWAS summary data from unrelated European ancestry participants adjusted for age, sex, genotyping chip, and within ancestry genetic principal components. TLNWL (UKB Field ID 20479) and TSH (UKB Field ID 20485) asked participants to respond “No,” “Yes, once,” or “Yes, more than once” to questions about thought/contemplation of self-harm. These items were dichotomized into “no” and “yes” for GWAS [15]. ASH (UKB Field ID 20480) and AS (UKB Field ID 20483) asked participants to respond with “no” or “yes” to questions about ASH behavior. These four items also were aggregated into a single ordinal trait termed “suicidality” such that participants responding “no” to all four questions were assigned “0” and each “yes” increased the participants’ suicidality score up to 4 (most severe). UKB participants with death-by-suicide ICD codes X60-X84 (classified as intentional self-harm) were excluded from GWAS. Further description of these variables has been published previously [15, 19, 20].

The use of UKB individual-level data has been conducted through the application reference number 58146. UKB has approval from the North West Multi-center Research Ethics Committee as a Research Tissue Bank (RTB) approval. This approval means that researchers do not require separate ethical clearance and can operate under the RTB approval.

### Linkage disequilibrium score regression (LDSC)

LDSC was used to estimate the *h*^*2*^-SNP of the *S*-factor based on the 1000 Genomes Project European ancestry reference panel. Stratified-LDSC (S-LDSC) was implemented in GenomicSEM for >51 genomic annotations (baseline annotation v2.2 with flanking and continuous annotations excluded) related to allele frequency strata, genomic conservation, evolutionary selective pressure, epigenomic regulatory sites, etc [21–24]. The major histocompatibility complex region was excluded from these analyses due to its complex linkage disequilibrium structure.

LDSC also was used to estimate the genetic correlation (*r*_*g*_) between suicidality and the *S*-factor relative to various suicide-associated traits and risk factors including large GWAS of psychiatric disorders. These were: TLNWL, TSH, ASH, and AS reported by Strawbridge, et al. [15], suicide attempt among bipolar disorder, schizophrenia, and major depression cases from Mullins, et al.; [14] psychiatric disorder GWAS from the Psychiatric Genomics Consortium including ADHD [25], anorexia nervosa [26], obsessive compulsive disorder [27], schizophrenia [28], Tourette syndrome [29], and the MVP including problematic alcohol use [30], posttraumatic stress disorder and its symptom domains [11], broad depression [13], and generalized anxiety disorder [31]; personality domains from the Genetics of Personality Consortium including extraversion, agreeableness, conscientiousness, openness [32]; and other related variables from the Social Science Genetic Association Consortium including subjective well-being [33], neuroticism [33], risky behavior [34], risk tolerance [34], cognitive performance [35], education years [35], and educational attainment [35]. We corrected the genetic correlation analysis for multiple testing using a false discovery rate of 5% to account for the known genetic and phenotypic overlap between the traits selected for this analysis.

### Effect size distribution

The R package GENESIS [36] was used to estimate common variant effect size distributions for suicidality and the S-factor. Effect size distributions are characterized by three statistics: π_c_ describes the proportion of susceptibility SNPs, σ^2^ describes the variance in effect size for non-null SNPs, and α describes residual effects not captured by the variance of effect-sizes such as population stratification, underestimated effects of extremely small effect size SNPs, and/or genomic deflation. We performed 2-component modeling to specify the effect of non-null SNPs [24, 36, 37]. GWAS data were filtered to (i) include only HapMap3 SNPs (excluding the major histocompatibility complex due to its complex linkage disequilibrium structure), (2) exclude SNPs with Z^2^ > 80, and (3) exclude SNPs with effective samples sizes <0.67-times the 90th percentile of the total sample.

We also included GWAS data for height [38] and broad depression [13]. Height was used as a model trait with broad effect size distribution representing a relatively large proportion of non-null SNPs with relatively large effect sizes. MDD was used as a model trait with narrow effect size distribution representing a relatively small proportion of non-null SNPs with relatively small effect sizes.

### GTEx v8 tissue enrichment

Tissue transcriptomic profile enrichment was evaluated using Multi-marker Analysis of GenoMic Annotation (MAGMA) [39]. To identify tissue effects of each phenotype, gene-property analyses were applied with Functional Mapping and Annotation (FUMA) to test relationships between tissue-specific gene expression profiles and disease-gene associations [40].

### Transcriptome-wide association studies

Summary-based transcriptome-wide association studies (TWAS) of suicidality and the S-factor were performed using the GTEx v8 TWAS expression weights for cerebellar hemisphere, cerebellum, hippocampus, and hypothalamus. Gene expression weights for 6091 features of the cerebellar hemisphere are estimated in 157 individuals, for 7272 features of the cerebellum are estimated in 188 individuals, for 3547 features of the hippocampus is estimated in 150 individuals, and for 3543 features of the hypothalamus are estimated in 156 individuals. FUSION [41] was used to perform TWAS of suicidality and all four S-factor indicators. TWAS of the S-factor was performed using gSEM and the TWAS summary association data from FUSION for each S-factor indicator. Multiple testing correction was applied using a Bonferroni threshold per tissue (*P* < 8.21 × 10^−6^ = 0.05/6091 genes for cerebellar hemisphere; *P* < 6.88 × 10^−6^ = 0.05/7272 genes for cerebellum; *P* < 1.41 × 10^−5^ = 0.05/3547 genes for hippocampus; *P* < 1.41 × 10^−5^ = 0.05/3543 genes for hypothalamus). RHOGE was used to estimate the genome-wide genetic correlation between suicidality and the S-factor as a function of predicted *cis* gene expression effects on each trait [42].

### Cross-ancestry translation of European-ancestry polygenic risk

We applied cross-ancestry polygenic scoring (PGS) in the UKB (Application Number 58146) to evaluate how findings from individuals of European ancestry extend to diverse communities. We derived suicidality in five additional groups as described previously: African (AFR; *N* = 876), Admixed American (AMR; *N* = 256), Central/South Asian (CSA; *N* = 1106), East Asian (EAS; *N* = 599), and Middle Eastern (MID; *N* = 269) [15, 20]. Ancestry groups were defined using a random forest classifier based on genetic principal components relative to a combined reference panel from the 1000 Genomes Project Phase III and the Human Genome Diversity Project. This procedure is described in detail at the Pan-Ancestry UKB web-page: https://pan.ukbb.broadinstitute.org/docs/qc.

Suicidality and *S*-factor polygenic scores (PGS) with continuous shrinkage were calculated for individuals from each ancestry group using PRS-CS [43]. PRS-CS is a Bayesian polygenic prediction method that imposes continuous shrinkage priors on SNP effect sizes. LD-independent SNPs were selected based on the UKB European ancestry reference panel. We further required that each SNP have a minor allele frequency >5% in the target ancestry group. Generalized linear models associating suicidality with suicidality and S-factor polygenic scores included age, age^2^, sex × age, sex × age^2^, and ten within-ancestry genetic principal components. We also tested the association of polygenic scores with each suicidality indicator trait (note that AS had too few observations to test) and depression (endorsement of either UKB Field ID 2090 or 2100) [44], total neuroticism score (inverse rank normalized, UKB Field ID 20127), and standing height (in centimeters; UKB Field ID 50).

## Results

### The *S*-factor structure

**Fig. 1:**
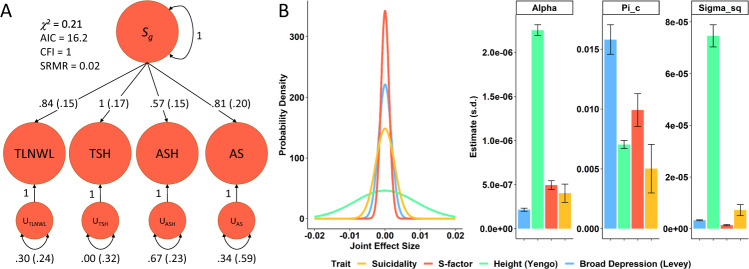
Factor structure and genetic architecture of the *S*-factor. **A** The four thoughts and behaviors associated with death by suicide (*thought life not worth living* = TLNWL, *thoughts of self-harm* = TSH, *actual self-harm* = ASH, and *attempted suicide* = AS) fit a single common factor (*S*-factor fit statistics are shown in the top left corner). The TSH indicator loading was constrained to 1; Table S3 shows indicator loadings before and after Heywood-case correction. **B** Genetic effect size distribution and associated statistics of the S-factor relative to suicidality using height and broad depression as comparative traits with relatively large and small proportions of relatively high effect size SNPs, respectively. AIC = Akaike information criterion, CFI = comparative fit index, SRMR = standardized root mean square residual, “pi_c” (π_c_) = proportion of susceptibility SNPs, “sigma_sq” (σ^2^) variance in effect size for non-null SNPs, and “alpha” (α) = residual effects not captured by the variance of effect-sizes (e.g., population stratification).

Using GenomicSEM, we constructed a common factor model that we refer to as the *S*-factor as it reflects the multivariate effects of the thoughts and behaviors proceeding death by suicide. We considered four indicators in the *S*-factor: *thought life not worth living* = “TLNWL,” *thoughts of self-harm* = “TSH,” *actual self-harm* = “ASH,” and *attempted suicide* = “AS.” TLNWL, TSH, and ASH had significant non-zero *h*^*2*^ (Table S1) but AS did not (*h*^*2*^ = 3.34%, *P* = 0.099) so we considered two *S*-factor models: model-1 included TLNWL, TSH, ASH, and AS (Fig. 1A) and model-2 included only TLNWL, TSH, and ASH. Genetic correlations (*r*_*g*_s) for each pair of indicators are shown in Table S2. Model-1 (*χ*^2^(2) = 0.21, AIC = 16.21, CFI = 1.00, SRMR = 0.024) had superior fit statistics relative to model-2 (*χ*^2^(2) = 3.14, AIC = 19.14, CFI = 0.999, SRMR = 0.025) and was chosen for all subsequent *S*-factor analyses (Table S3). TSH had the highest genetic correlation with suicidality (*r*_*g*_ = 0.989, *P* = 3.62 × 10^−76^) and was the indicator most strongly loaded onto the *S*-factor (standardized loading = 1 ± 0.17).

### Genetic architecture of the *S*-factor

There were no loci associated with the *S*-factor at the level of genome-wide significance (GWS, *P* < 5 × 10^−8^; Fig. 1). All three suicidality loci from Strawbridge, et al. [15]. were nominally replicated in the *S*-factor GWAS (Table S4) with no significant difference in effect size between the two studies.

We quantified several metrics of genome-wide polygenicity (Fig. 1B, C and Table S5A) using GENESIS [36]. Relative to suicidality (π_c_ = 0.005 ± 0.002), the *S*-factor has a significantly higher proportion of non-null susceptibility SNPs (π_c_ = 0.010 ± 0.001, P_diff_ = 0.045; Fig. 1C and Table S5A). However, the suicidality effect size distribution is broader than that of the *S*-factor (Fig. 1B), suggesting that suicidality SNPs effect sizes are generally greater and may require smaller samples sizes to detect by GWAS [15]. When projected sample sizes reach 500,000 (Table S5B) the *S*-factor GWAS is estimated to remain uninformative while the suicidality GWAS should yield 60 GWS SNPs (95% CI: 15–139). At projects of 1-million individuals, the *S*-factor remains relatively uninformative (4 GWS SNPs, 95% CI: 0–18). Only when projected to 5-million individuals does the *S*-factor GWAS become an informative source of associated loci (539 GWS SNPs, 95% CI: 208–1117); however, the suicidality GWAS remained the more lucrative study in terms of susceptibility loci discovered at all projected sample sizes (Table S5B and S5C).

### Heritability comparisons

The *h*^*2*^-SNP of the *S*-factor was 2.54% (*P* = 1.72 × 10^−12^) which is significantly lower than the *h*^*2*^-SNP for a pooled suicidality phenotype (*h*^*2*^-SNP = 7.6%, *P*_diff_ = 4.78 × 10^−13^) [15]. Though different with respect to phenotypic variance explained by common genetic variation, the *r*_*g*_ between the *S*-factor and suicidality is almost perfect (*r*_*g*_ = 0.996, *P* < 9.21 × 10^−308^).

**Fig. 2:**
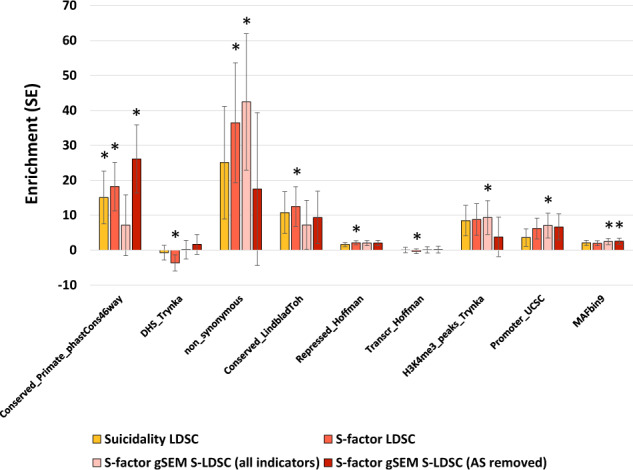
Genomic annotation enrichment. Enrichment of 9 genomic annotations at least nominally enriched (asterisks indicate *P* < 0.05) in the GWAS of suicidality and/or the *S*-factor. Genomic annotations have been described previously [21, 22, 24]. All genomic annotation enrichments are provided in Table S6. Definitions: Conserved_Primate_phastCons46way = genomic regions conserved across primate species [21], DHS_Trynka = DNase I hypersensitive sites reflecting aggregated ENCODE and Roadmap data [59], non_synonymous = non-synonymous SNPs, Conserved_LindbaldToH = genomic regions conserved in mammals [60], Repressed_Hoffman = CTCF repressed chromatin elements intersected from six cell lines from Hoffman, et al. [61], Transcr_Hoffman = CTCF transcribed elements across six cell lines from Hoffman, et al. [61], H3K4me3_peaks_Trynka = Cell-type-specific H3K4me1, H3K4me, and H3K9ac data from Roadmap [59], Promoter_UCSC = genomic regions contains in gene promoter elements [62, 63], MAFbin9 = loci with minor allele frequencies within (37.73; 43.87] in the 1000 Genomes Project Phase 3 and (37.66; 43.76] in UK 10k [64].

S-LDSC was applied to quantify the overlap between genomic annotation contributions to *h*^*2*^-SNP in the suicidality and *S*-factor GWAS. We partitioned the *h*^*2*^-SNP of the *S*-factor three ways: with LDSC, with gSEM S-LDSC using all indicators, and with gSEM S-LDSC removing the least well-powered indicator (AS). This approach permitted comparison across methods for robust detection of enriched genomic categories using the various strengths of each method (e.g., gSEM S-LDSC reports a Z-smooth value quantifying the degree of smoothing applied to the data with clear guidelines for enrichment interpretation given these values). The most consistently enriched annotation described sites conserved across primates as measured by PhastCons 46-way alignment (*converved_primate_phastcons46way*, mean enrichment = 19.80 ± 5.68, Fig. 2 and Table S6). Regardless of trait definition or partitioning method applied, there were no significant differences in genomic enrichment (*P* ≥ 0.147).

### Genetic correlation

We next compared *r*_*g*_ estimates of suicidality and the *S*-factor relative to 32 mental health traits (Table S7), including other genetic assessments of the thoughts and behaviors associated with death by suicide. Because of the high genetic overlap between the S-factor and suicidality, the *r*_*g*_ estimates with other mental health traits were nearly identical (adjusted *R*^*2*^ = 0.984, *P* = 8.55 × 10^−188^). Though not significantly different, the largest magnitude of difference in *r*_*g*_ estimates stems from comparisons with TLNWL (*r*_*g*_ with suicidality = 0.870, *r*_*g*_ with *S*-factor = 0.941, *P*_diff_ = 0.284).

### Comparison of brain region transcriptomic effects

Consistent with prior findings from this study, tissue transcriptomic profile enrichments for suicidality and the *S*-factor are highly correlated (*R*^*2*^ = 0.861, *P* = 3.265 × 10^−34^). Though the *S*-factor GWAS yielded two significant tissue transcriptomic profile enrichments, there was no difference in effect size for the enrichments derived from suicidality or the *S*-factor. Gene expression weights from four brain tissues were used for TWAS comparisons of suicidality and the S-factor due to their significant enrichments (*P* < 0.05) in the suicidality GWAS: cerebellar hemisphere, cerebellum, hippocampus, and hypothalamus (Table S8).

Though no gene reached GWS in any of the TWAS performed (Table S9), the genetic correlation between genetically predicted gene expression effects underlying suicidality and the S-factor in each tissue was high: *ρ*_GE_ = 0.990, *P* = 1.31 × 10^−296^ considering cerebellar hemisphere weights; *ρ*_GE_ = 0.991, *P* < 9.21 × 10^−308^ considering cerebellum weights; *ρ*_GE_ = 0.991, *P* = 8.96 × 10^−175^ considering hippocampus weights; *ρ*_GE_ = 0.992, *P* = 1.81 × 10^−193^ considering hypothalamus weights. The most significant protein-coding gene expression effects discovered from the more powerful suicidality TWAS were *RBM26* in the cerebellar hemisphere (suicidality *Z* = 3.95, *P* = 7.53 × 10^−5^; S-factor *Z* = 2.78, *P* = 0.005) and *COLQ* in the hippocampus (suicidality Z = −3.90, *P* = 9.53 × 10^−5^; S-factor *Z* = 3.12, *P* = 0.001).

### Cross-population polygenic scoring

**Fig. 3:**
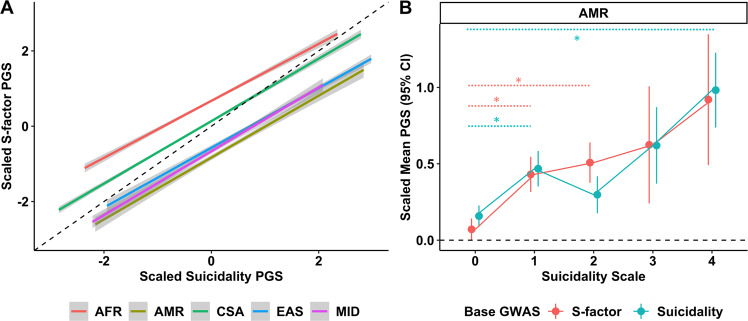
Cross-population polygenic scoring. **A** Linear relationships between suicidality and *S*-factor polygenic scores estimated using European ancestry GWAS summary association data in five diverse ancestry groups. **B** Portability of suicidality and *S*-factor polygenic scores into Admixed American suicidality data from the UK Biobank. Asterisks indicate a significant difference in polygenic score relative to the control (suicidality scale = 0).

Using SNP effect sizes estimated from large European-ancestry GWAS, PGS for suicidality and the S-factor in diverse ancestries were highly correlated (minimum *Pearson’s r* = 0.726, *P* = 3.25 × 10^−214^ in AFR; maximum *Pearson’s r* = 0.824, *P* = 1.92 × 10^−121^ in MID; Fig. 3A). PGS for suicidality associated with suicidality (*R*^*2*^ = 11.1%, *P* = 0.017), TLWNL (Nagelkerke’s *R*^*2*^ = 8.56%, *P* = 0.009), and ASH (Nagelkerke’s *R*^*2*^ = 14.3%, *P* = 0.034) in the AMR population; PGS for the *S*-factor associated with TLNWL in AMR (Nagelkerke’s *R*^*2*^ = 7.48%, *P* = 0.045) and with ASH in MID (Nagelkerke’s *R*^*2*^ = 7.71%, *P* = 0.046; Table S10 and Fig. 3B). PGS for the *S*-factor also associated with neuroticism scores and depression in several diverse populations but the analogous test with suicidality PGS were generally not significant. However, there were no differences in effect size for the PGS regardless of the GWAS used to train them. In the CSA population, suicidality (Nagelkerke’s *R*^*2*^ = 4.39%, *P* = 0.048) and *S*-factor (Nagelkerke’s *R*^*2*^ = 4.31%, *P* = 0.048) PGS both associated with depression (Table S10). As a null control, we observed no relationship between PGS for suicidality or *S*-factor and height.

## Discussion

The use of continuous or ordinal phenotypes for GWAS of psychiatric disorders and related psychopathologies has proven a powerful way to identify risk loci and related biological pathways underlying common diagnoses [11–13]. This approach has been previously applied to studies of suicidality that apply an equal weight to each questionnaire item [15]. In other words, regardless of the relationship between questionnaire items or the sensitivity of those items for an outcome, each contributed equally to the outcome. We compared this approach to capturing a suicidality phenotype to gSEM-derived *S*-factor which explicitly considers the relationship between each indicator variable in the model.

All four suicidal thoughts and behaviors significantly loaded onto the observed *S*-factor. Relative to epidemiological data supporting AS as the leading predictor of future death by suicide, the genetic component of the *S*-factor was most associated with TSH. AS was the least heritable indicator in this study but removing AS from the *S*-factor reduced model fit suggesting that this indicator is relevant on the genetic level. Therefore, AS may require larger sample sizes to become highly relevant to the *S*-factor structure. Instead, we suspect the major contribution of TSH to the *S*-factor stem from a balance between (i) heritability and power and (ii) increased specificity for suicidal thoughts and behaviors. TLNWL was the most powerful indicator GWAS but has previously shown extremely high phenotypic and genetic correlation with MDD (*r*_*g*_ = 0.46) and neuroticism (*r*_*g*_ = 0.56) [15] and is even a component of the Personal Health Questionnaire 9-item measure of depressive symptoms [45].

The potential for risk locus discovery in GWAS of suicidality and the *S*-factor produced the most notable differences. Though the *S*-factor had a significantly larger number of non-null SNPs, these loci required substantially larger sample sizes to detect their relatively small effect sizes. It would require an estimated 5-million individuals with a similar prevalence of S-factor indicator endorsement to yield GWS SNPs in numbers already surpassed in GWAS of correlated traits like MDD [13] and schizophrenia [28]. The phenotypically aggregated suicidality item had an earlier return on investment producing hundreds of GWS SNPs with cohorts ranging from 500-thousand and 1-million participants. We hypothesize that this difference between suicidality and the *S*-factor future return on investment in part reflects aggregated effects of low *S*-factor *h*^*2*^-SNP estimates, relatively high heterogeneity among the primary S-factor indicator traits, and lack of non-null SNPs for the *S*-factor from which to model the non-null SNP distribution and future projections. Furthermore, suicidality had a significantly higher *h*^*2*^ estimate suggesting this phenotype is more informative for inferring relevant biology through downstream in silico analyses. This is reinforced by a lack of significant differences in SNP effect sizes between the GWAS of suicidality and the *S*-factor and may stem from utilizing a linear regression for the suicidality GWAS relative to the individual logistic regressions performed for each of the UKB items contributing to the *S*-factor GWAS. It should also be noted that the reduced *h*^*2*^ of the *S*-factor may arise from its attempt to parse suicidal ideation and self-harm ideation and therefore may not model the distribution of thoughts and behaviors as expected. In other words, ASH and AS may be too poorly represented in the model due to *h*^*2*^, sample size, or substantially different genetic features that cannot be accommodated by our hypothesized one-factor model. The application of linear models to an ordinal trait like suicidality complicates the interpretation of how GWS loci increase or decrease risk for such thoughts and behaviors due to the forced linear relationship in a non-linear space. An ordinal-trait aware SNP-phenotype regression may better model the skew in ordinal data commonly observed for traits ascertained through biobank surveys [46]. Future work is necessary to understand the benefit of explicitly modeling ordinal data in genotype-phenotype associations of suicidal thoughts and behaviors. For example, to better understand how GWS loci increase or decrease risk for suicidality in a potentially non-linear fashion across suicidality categories.

To our knowledge, the enrichment of SNP-annotations related to conserved genomic regions is the first of its kind for traits along the suicidality spectrum but was consistent across approaches. The magnitude of enrichment also was consistent with those reported for MDD [44] and across psychiatric disorders more broadly [47]. In the context of loci identified in GWAS of major depression, genes found in conserved regions of the genome were part of networks relevant for organismal development and function across the lifetime such as synaptic function and brain development [48, 49]. These enrichments in major depression GWAS suggest a rich interaction between genetic factors and the environment that has been empirically demonstrated for suicidality and select environments related to stress [20], substance use [19], and depression [50].

Several tissue transcriptomic profiles from brain regions were nominally enriched in the GWAS of suicidality but the *S*-factor GWAS was underpowered to detect similar enrichments. We further tested these enrichments using a TWAS approach in the cerebellar hemisphere, cerebellum, hippocampus, and hypothalamus. There were extremely high correlations between the suicidality and *S*-factor regardless of tissue; however, these relationships were estimated using only *cis*-elements only [42]. We therefore cannot rule out the contribution of *trans*-regulatory elements to gene expression differences between suicidality and the S-factor.

Though no gene reached genome-wide significance, the two most significantly associated genes harbor interesting functional relevance worth discussing. *RBM26* was associated with schizophrenia in a recent study but only at a level of suggestive significance (*P* = 3.41 × 10^−7^) [51]. Within the first decade of diagnosis, people who suffer from schizophrenia are at the highest risk for suicidality, with a total suicide rate of 10% [52]. Though there are several factors contributing to decreased life expectancy in schizophrenics, suicide is the largest one. *COLQ* is associated with cardiovascular traits such as resting heart rate (*P* = 1.59 × 10^−12^) [53]. An increased baseline resting HR of 10 beats per minute increased the suicide rate by 19% in one study [54]. Though accounting for many essential covariates such as smoking status, sex, body mass index, stress, depressed mood, and use of psychotropic medications, this study, to our knowledge, failed to account for population stratification and socioeconomic status. Though not directly related, *RBM26* and *COLQ* may have pleiotropic links to suicidality. Further research is required to untangle the cause-effect relationships between these potential risk factors and the severity of suicidal thoughts and behaviors.

We performed a cross-ancestry PGS analysis with respect suicidality and the *S*-factor which showed (i) limited portability to the AMR population with respect to suicidality measures, (ii) strong portability to the CSA population with respect to the *S*-factor indicator TLNWL, (iii) limited association between suicidality or *S*-factor PGS with other mental health outcomes associated with death by suicide, and (iv) lack of association between PGS and height, an unrelated trait. These findings are in line with the limited translation of EUR-derived PGS in existing cross-ancestry studies of transdiagnostic mental health characteristics [55]. Of note from our study is the relatively large variance explained by the PGS in some of the diverse ancestries tested. This may partially be attributed to methodological benefits of a Bayesian approach but may also suggest consistent genetic architectures of suicidality across populations. Large studies are ongoing to learn about the genetic components of suicidal thoughts and behaviors in diverse ancestries and will permit deeper investigation of the S-factor and suicidality.

We demonstrated that the genetic and transcriptomic signatures of suicidality and the *S*-factor strongly overlap but our study has some limitations to consider. First, this body of work relies on large studies of European ancestry individuals clustered into this grouping using genetic principal components. Our results support limited translation of these results to diverse populations. It is well documented that these communities experience (i) social and cultural stigma surrounding suicidal thoughts, behaviors, and associated death [56, 57] and (ii) vastly different face-to-face interactions with the healthcare system [58]. For these reasons, our findings may not translate across diverse communities that disproportionately experience these thoughts and behaviors. Dedicated community outreach, sample recruitment, and educational programs are necessary to perform robust studies of suicidal thoughts and behaviors in other contexts, especially as they relate to community stressors that may interact with underlying genetic factors. Second, the UKB is limited by the potential for recruitment, survivor, and recall bias as this cohort is generally older, wealthier, and better educated than a general community sampling. This cohort may therefore be depleted for the more extreme ends of the suicidality spectrum and better reflect milder suicidality ratings than those from a more representative sampling. Finally, death by suicide and the preceding thoughts and behaviors routinely co-occur with psychiatric diagnoses. The GWAS used to construct the *S*-factor did not take into consideration the effects of co-morbid depression, anxiety, chronic pain, or other necessary experiences or diagnoses. There is evidence that genetic findings related to suicidality are independent of psychiatric diagnoses [1, 6, 14, 15] but it remains unclear how best to account for these variables to make discoveries with as much specificity for suicidal thoughts and behaviors as possible. Finally, we used dichotomous measures of suicidality as indicator traits to construct the *S*-factor. The construction of these items (e.g., the suicidal and non-suicidal self-injury in the ASH phenotype) may underestimate heritability and its contribution to the *S*-factor structure.

Despite these limitations, this study empirically investigated the differences and similarities between definitions of suicidality. Across all analyses presented, the phenotypically-aggregated suicidality item was more statistically powerful and informative than the *S*-factor for downstream in silico characterization of the biology underlying this complex trait. In conclusion, our study informs one path forward for the analysis of participant responses related to thoughts and behaviors associated with death by suicide. By aggregating multiple informative items into a suicidality phenotype, studies are likely to generate more information about suicide biology compared to individual binary items or a genetically-defined *S*-factor.

## Supplementary information

Supplementary Data Descriptions

Supplementary Data Tables

## Data Availability

*S*-factor summary statistics can be accessed via Zenodo 10.5281/zenodo.7433120.
