# Supplementary material for: Functional and molecular characterization of suicidality factors using phenotypic and genome-wide data
Source: Mol Psychiatry. 2023 Jan 6;28(3):1064–71. doi: 10.1038/s41380-022-01929-5 (PMC10005939; doi:10.1038/s41380-022-01929-5)
Supplement: Supplementary file 1 — Supplementary Data Descriptions [file 41380_2022_1929_MOESM1_ESM.docx]

# **SUPPLEMENTARY MATERIAL**

**Table S1.** SNP-based heritability estimates for the four indicator traits used to derive the S-factor.

**Table S2.** Genetic correlation estimates (s.e.) for the four indicator traits used to derive the S-factor.

**Table S3.** Structure of the S-factor using two separate models. Model 1 includes attempted suicide in the factor structure while model 2 removes it due to lack of significant SNP-based heritability estimate.

**Table S4.** Comparison of effect sizes for three prior suicidality SNPs discovered in Strawbridge, et al.^15^

**Table S5.** Summary of effect size distribution comparisons between suicidality and the S-factor. Table A compares effect size distribution metrics; B compares the projected number of SNPs recovered from increasing sample sizes of each trait; C compares the projected variance explained from increasing sample sizes of each trait.

**Table S6.** Partitioned heritability results for enrichment of genomic annotations. Yellow boxes denote significant enrichments.

**Table S7.** Genetic correlation estimates for 32 mental health traits related to suicidality and the S-factor.

**Table S8.** Tissue transcriptomic profile enrichment for suicidality and the S-factor using GTEx v8.

**Table S9.** TWAS results for suicidality and S-factor in four brain tissues (GTEx v8): cerebellar hemisphere, cerebellum, hippocampus, and hypothalamus.

**Table S10.** Results of cross-population polygenic scoring. Yellow highlight indicates a significant result in either the suicidality test or the S-factor test. Yellow highlight plus red text indicates a significant result in both tests.
